# An Intelligent Real-Time System for Sentence-Level Recognition of Continuous Saudi Sign Language Using Landmark-Based Temporal Modeling

**DOI:** 10.3390/s26051652

**Published:** 2026-03-05

**Authors:** Adel BenAbdennour, Mohammed Mukhtar, Osama Almolike, Bilal A. Khawaja, Abdulmajeed M. Alenezi

**Affiliations:** Department of Electrical Engineering, Faculty of Engineering, Islamic University of Madinah, Madinah 42351, Saudi Arabiamoeba0702@gmail.com (M.M.);

**Keywords:** sign language, Saudi Sign Language, sentence-level recognition, Bidirectional Long Short-Term Memory, large language model, deep learning, assistive technologies, disability, temporal modeling, artificial intelligence

## Abstract

A persistent challenge for Deaf and Hard-of-Hearing individuals is the communication gap between sign language users and the hearing community, particularly in regions with limited automated translation resources. In Saudi Arabia, this gap is amplified by the reliance on Saudi Sign Language (SSL) and the scarcity of real-time, sentence-level translation systems. This paper presents a real-time system for sentence-level recognition of continuous SSL and direct mapping to natural spoken Arabic. The proposed system operates end-to-end on live video streams or pre-recorded content, extracting spatio-temporal landmark features using the MediaPipe Holistic framework. For classification, the input feature vector consists of 225 features derived from hand and body pose landmarks. These features are processed by a Bidirectional Long Short-Term Memory (BiLSTM) network trained on the ArabSign (ArSL) dataset to perform direct sentence-level classification over a vocabulary of 50 continuous Arabic sign language sentences, supported by an idle-based segmentation mechanism that enables natural, uninterrupted signing. Experimental evaluation demonstrates robust generalization: under a Leave-One-Signer-Out (LOSO) cross-validation protocol, the model attains a mean sentence-level accuracy of 94.2%, outperforming the fixed signer-independent split baseline of 92.07%, while maintaining real-time performance suitable for interactive use. To enhance linguistic fluency, an optional post-recognition refinement stage is incorporated using a large language model (LLM), followed by text-to-speech synthesis to produce audible Arabic output; this refinement operates strictly as post-processing and is not included in the reported recognition accuracy metrics. The results demonstrate that direct sentence-level modeling, combined with landmark-based feature extraction and real-time segmentation, provides an effective and practical solution for continuous SSL sentence recognition in real-time.

## 1. Introduction

Sign language [1,2] is the primary means of communication for millions of deaf and hard-of-hearing individuals worldwide. In the Arab region, Arabic Sign Language (ArSL) includes national dialects; in Saudi Arabia, Saudi Sign Language (SSL) is the local dialect used in daily communication. Despite its importance, effective interaction between sign language users and hearing individuals remains limited, primarily due to the low prevalence of sign language proficiency among the general population. This communication gap affects access to education, public services, employment, and routine social interaction, creating persistent barriers to full societal participation.

Existing solutions for bridging this gap, such as human interpreters or text-based communication, are often impractical for spontaneous, real-time interaction. Human interpretation is costly and not always available, while text-based alternatives lack natural conversational flow and immediacy. These limitations have motivated increasing interest in automated sign language recognition (SLR) and translation systems, particularly with the recent progress in computer vision and deep learning. However, many existing approaches focus on isolated sign or gloss recognition and rely on explicit segmentation or intermediate annotations, which are difficult to obtain and poorly suited to continuous, real-world signing scenarios.

Continuous SLR presents distinct challenges due to the absence of clear boundaries between signs, variability in signing speed, and inter-signer differences in motion and execution style. Addressing these challenges requires models capable of capturing long-range temporal dependencies while operating under real-time constraints. Landmark-based representations extracted from RGB video offer an attractive solution, as they provide compact, illumination-robust features that abstract away appearance variability while preserving essential motion information.

In this work, we present an intelligent real-time system for sentence-level recognition of continuous ArSL. Throughout this paper, SSL denotes the target deployment context in Saudi Arabia, while experimental evaluation is conducted on the publicly available ArabSign continuous Arabic Sign Language (ArSL) dataset. The system operates end-to-end, transforming live or pre-recorded video into spoken Arabic output. Visual input is processed using the MediaPipe Holistic framework to extract hand and body landmarks, which are temporally segmented using an idle-based mechanism that enables natural, uninterrupted signing. A Bidirectional Long Short-Term Memory (BiLSTM) network performs direct sentence-level classification over a predefined vocabulary of fifty ArSL sentences derived from the ArabSign dataset. In this paper, the term translation refers to mapping continuous sign language input to a predefined sentence inventory followed by spoken Arabic rendering, rather than open-vocabulary sequence-to-sequence sign language sentence-level translation. To enhance usability and linguistic fluency, the recognized sentence may be refined using a large language model (LLM) before being converted into speech by a text-to-speech (TTS) module. All post-recognition components operate asynchronously, preserving real-time performance of the recognition pipeline.

The proposed approach emphasizes direct sentence-level modeling rather than gloss-based decomposition, thereby avoiding cumulative segmentation and annotation errors and aligning more closely with realistic deployment conditions. This distinction is particularly important for Arabic sign languages, where strong co-articulation causes adjacent signs to overlap temporally, making gloss-level boundary detection inherently noisy in continuous signing streams. Experimental evaluation is conducted under a signer-independent protocol to assess generalization to unseen individuals, and real-time performance is measured to ensure interactive usability.

The main contributions of this work are as follows:

- A real-time end-to-end system for continuous SSL recognition, operating directly at the sentence level without reliance on gloss annotations or manual segmentation.
- An idle-based temporal segmentation strategy that enables automatic detection of sentence boundaries in continuous signing streams under natural signing behavior.
- A landmark-based BiLSTM sentence classification framework trained and evaluated on the ArabSign dataset under a signer-independent protocol, comprising fifty sentence classes.
- An integrated post-recognition refinement and speech synthesis pipeline that enhances linguistic fluency; this module operates strictly as post-processing and is not included in any reported recognition accuracy metrics.

The remainder of this paper is organized as follows: Section 2 provides a review of related work in automated SLR and translation, documenting the transition from handcrafted pipelines to deep learning-based spatiotemporal models. Section 3 describes the ArabSign dataset, including its characteristics, the continuous signing behavior it captures, and the landmark-based feature extraction process using the MediaPipe framework. Section 4 details the proposed methodology, outlining the system overview, the idle-based segmentation mechanism, the BiLSTM-based sentence classification network, and the optional post-recognition refinement stage. Section 5 presents the experimental setup, covering the training protocols (including the Leave-One-Signer-Out cross-validation), evaluation metrics, and implementation details for both training and real-time deployment. Section 6 reports the experimental results, including training dynamics, robustness analysis of the segmentation parameters, and a performance comparison of different recurrent architectures under signer-independent evaluation. Section 7 provides a discussion of the findings, validates the use of direct sentence-level modeling, and analyzes the system’s performance from a deployment perspective. Finally, Section 8 concludes the paper and suggests directions for future work.

## 2. Literature Review

Automatic SLR and translation have been extensively studied as assistive technologies aimed at bridging communication gaps between Deaf and Hard-of-Hearing communities and the hearing population. Recent surveys document the rapid transition from handcrafted feature pipelines toward deep learning-based spatiotemporal models, while also emphasizing persistent challenges such as signer variability, continuous temporal structure, limited annotated corpora, and the trade-off between recognition accuracy and deployability under real-time constraints [1,2]. Early large-vocabulary studies on continuous sign language recognition (CSLR) established the foundational problem formulation, demonstrating that realistic recognition systems must operate on unsegmented temporal streams and generalize across multiple signers [3]. These works highlighted the inherent complexity of continuous recognition compared to isolated sign classification and motivated subsequent advances in temporal modeling.

With the adoption of deep neural networks, end-to-end approaches have significantly advanced both CSLR and sign language sentence-level translation. Encoder–decoder architectures for neural sign language sentence-level translation mapped visual sign sequences to spoken-language text, often relying on intermediate gloss representations to stabilize alignment and improve translation quality [4]. Transformer-based models further strengthened this paradigm by capturing long-range temporal dependencies and jointly optimizing recognition and translation objectives within a unified framework, achieving strong performance on standard benchmarks [5]. Extending this line of high-capacity modeling, large-scale multimodal pre-training paradigms have leveraged curated sign–text corpora and joint pretext learning objectives to improve generalization across diverse sign language understanding tasks [6]. In parallel, research has explored strategies to mitigate data scarcity, including sign back-translation using monolingual text resources [7] and cross-modal knowledge distillation to enhance continuous recognition performance by transferring complementary information across modalities and representations [8]. Similarly, a hierarchical recurrent fusion framework has been proposed to address weakly supervised sequence-to-sequence sign language sentence-level translation, integrating adaptive temporal encoding with multimodal feature fusion to enhance sentence-level modeling under signer-independent evaluation [9]. Despite their effectiveness, such approaches are typically evaluated offline and rely on computationally intensive architectures that may not align with the latency and stability requirements of interactive, real-time systems.

A complementary and increasingly practical direction focuses on representation efficiency rather than architectural scale. Landmark-based and skeleton-based representations replace raw pixel inputs with structured keypoints capturing hand, body, and facial kinematics, reducing sensitivity to illumination, background clutter, and signer appearance while enabling more compact temporal modeling. General-purpose pose estimation frameworks enable reliable, real-time extraction of human keypoints from monocular RGB input [10], while production-oriented pipelines, such as MediaPipe, provide optimized, on-device solutions for landmark extraction suitable for real-time perception systems [11,12]. Building on such representations, skeleton-aware translation models explicitly encode kinematic structure, demonstrating that structured inputs can support competitive translation performance without relying on heavy visual encoders [13]. Graph-based multimodal sequential embedding strategies have further explored structured modeling of intra- and inter-modal correlations, demonstrating that relational representations can strengthen temporal abstraction in continuous sign language sentence-level translation settings [14]. Furthermore, preprocessing and normalization strategies for keypoint sequences have been shown to significantly affect downstream recognition and translation quality, motivating careful pipeline design when operating on landmark data [15,16].

Despite these methodological advances, ArSL remains comparatively under-represented in the CSLR literature, particularly at the sentence level. Many Arabic-focused systems address alphabets or isolated gestures, which simplifies temporal modeling but does not reflect natural signing dynamics. Representative examples include signer-independent isolated recognition frameworks [17] and skeleton-based systems operating on pre-segmented inputs [18]. Hybrid deep learning architectures combining convolutional backbones with recurrent and attention-based classifiers have also been explored for isolated sign recognition in small-scale Arabic settings [19]. While such approaches demonstrate the feasibility of deep learning and structured representations for Arabic signing, they do not address the continuous temporal boundary ambiguity inherent in dialogue-like signing. The ArabSign dataset constitutes a significant step forward by providing a multimodal benchmark for continuous Arabic SLR and enabling signer-independent evaluation at the sentence level [20], complementing earlier isolated-gesture resources such as ArASL presented in [21]. Recent work has begun to explore continuous Arabic SLR using staged processing strategies, such as predicting word counts prior to recognition; however, these approaches typically assume predefined segmentation cues and do not explicitly target real-time sentence-level deployment with stable latency characteristics [22].

Beyond recognition accuracy, the usability of sign language sentence-level translation systems depends on the linguistic quality and readability of the generated text. In related natural language processing research, systematic reviews of machine translation post-editing studies have documented trends and challenges in refining machine-generated outputs for human use, highlighting the evolving role of post-editing in translation workflows [23]. Recent evaluations demonstrate that instruction-tuned LLMs, even in zero-shot settings, can produce high-quality corrections with improved grammatical correctness and fluency on benchmark grammatical error correction tasks [24]. Within the sign language domain, gloss-free translation frameworks incorporating LLMs have been proposed to enhance text generation under low-resource conditions, demonstrating the potential of modular language-side refinement without altering the core visual recognizer [25]. Nevertheless, the integration of such refinement strategies into real-time Arabic CSLR pipelines remains largely unexplored.

In summary, existing literature reveals a clear trade-off between accuracy-driven architectures and deployment-oriented system design. High-capacity transformer models dominate offline benchmarks for CSLR and translation, whereas lightweight, landmark-based temporal models offer favorable efficiency and stability properties but have received comparatively less attention at the sentence level for ArSL. Moreover, most Arabic-focused studies either operate on isolated signs or assume pre-segmented inputs, leaving continuous temporal boundary handling and real-time behavior insufficiently addressed. These gaps motivate approaches that prioritize efficient landmark representations, continuous sentence-level modeling under signer-independent evaluation, and optional post-recognition linguistic refinement aligned with the practical requirements of interactive deployment.

## 3. Dataset

The experiments in this study are conducted on the ArabSign continuous ArSL dataset, designed for sentence-level recognition [20]. The dataset is designed to capture realistic continuous signing behavior, including natural pauses and signer variability, to reflect practical deployment conditions rather than isolated sign execution. The dataset comprises 9335 video samples representing 50 distinct ArSL sentences performed by six different signers. All recordings were processed as RGB video at 24 frames per second (FPS), resulting in a total dataset duration of approximately 10 h and 13 min.

Sentence segment lengths range from 48 to 189 frames, with a mean of 87 frames and a standard deviation of 24 frames. Variability is introduced by differences in signing speed and sentence complexity. It should be noted that this average reflects the effective sentence segment duration after idle-based boundary detection rather than the full raw recording length. Because recordings were captured as continuous streams, including short natural pauses before and after signing, non-signing idle frames are removed during segmentation. Consequently, the average frame count represents the active signing portion used for modeling rather than the entire recorded clip duration.

Recordings were conducted in an indoor environment with controlled background conditions to reduce visual clutter while preserving natural hand and facial motion. Signers were instructed to produce sentences continuously without manual start-stop cues, thereby allowing natural pauses between sentences. This design supports the evaluation of automatic idle-based sentence segmentation under realistic operating conditions. For each video frame, anatomical landmarks corresponding to the face, body pose, and both hands are extracted using the MediaPipe framework. While facial landmarks are extracted by the MediaPipe Holistic framework for visualization and future extensibility, the per-frame classifier input consists of 225 features derived from body pose (99) and hand landmarks (126), each represented using (x, y, z) coordinates. Landmark-based features are used in place of raw RGB inputs due to their compact representation and reduced sensitivity to illumination variation. Continuous signing streams are automatically segmented using an idle-based mechanism. Each detected sentence segment is subsequently normalized to a fixed temporal length of 80 frames through linear interpolation or uniform down-sampling, ensuring consistent input dimensionality for recurrent sequence modeling. For experimental evaluation, the dataset is partitioned using a signer-independent protocol such that samples from the same signer do not appear simultaneously in training and testing sets. This protocol prevents identity leakage and enables a realistic assessment of generalization to unseen signers. A summary of the dataset characteristics is provided in Table 1. The ArabSign dataset is publicly available and is used in this work under its original licensing terms, with all experiments conducted in accordance with the dataset’s intended research usage.

## 4. Methodology

This section presents the methodological foundations of the proposed real-time Arabic CSLR system. The description outlines the implemented pipeline and explains the processing stages and design choices required for sentence-level recognition from continuous signing streams under real-time constraints.

### 4.1. System Overview

**Figure 1:**
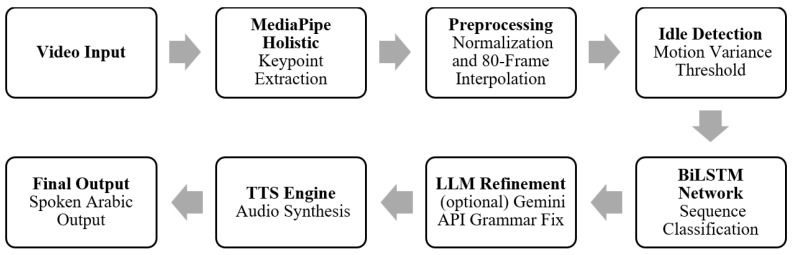
Overall architecture of the proposed real-time Arabic CSLR system, showing video acquisition, landmark extraction, idle-based segmentation, temporal normalization, BiLSTM classification, and post-recognition enhancement. The post-recognition refinement stage is optional and operates strictly as post-processing; it is not included in any reported recognition accuracy metrics.

The proposed system is designed as an end-to-end visual pipeline that transforms continuous RGB video streams into sentence-level Arabic text output in real-time. The overall architecture follows a modular design in which each stage performs a clearly defined function while maintaining strict latency constraints. This modularity supports interpretability and enables the behavior of each stage to be analyzed independently within the full pipeline. Unlike gloss-based or word-level SLR approaches, the system operates directly at the sentence level. This design reflects practical deployment expectations in which users require coherent sentence-level output rather than sequences of isolated signs. Consequently, the system must handle continuous input, implicit sentence boundaries, and temporal variability across executions. An overview of the complete processing pipeline, including video acquisition, landmark extraction, idle-based segmentation, temporal normalization, and sentence-level classification, is illustrated in Figure 1.

### 4.2. Recognition Pipeline

**Figure 2:**
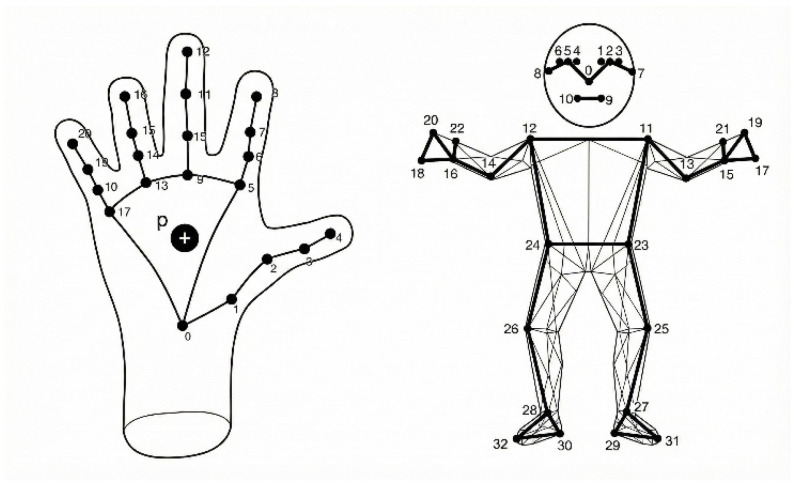
Illustration of the structured hand, face, and pose landmarks extraction.

Visual features are extracted using a landmark-based representation obtained from each RGB frame. The system uses structured landmarks extracted from the hands, face, and body pose, while the classifier input is constructed from hand and pose landmarks only, consistent with the 225-dimensional feature representation. Although facial landmarks are detected during extraction, they are excluded during feature selection, as the primary discriminative content for sentence-level SSL recognition in this design is captured by hand configuration and arm motion dynamics. Landmark features are used in place of raw RGB pixels due to their lower dimensionality and improved robustness to appearance variation and lighting changes, while still preserving kinematic information essential for sign dynamics. The structured hand, face, and pose landmarks extracted from each video frame are illustrated in Figure 2.

**Figure 3:**
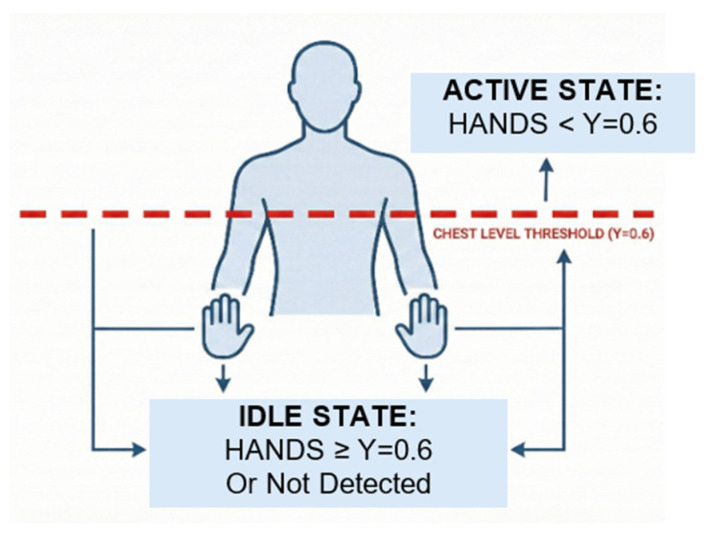
Idle-based segmentation to detect sentence boundaries in continuous signing streams.

**Figure 4:**
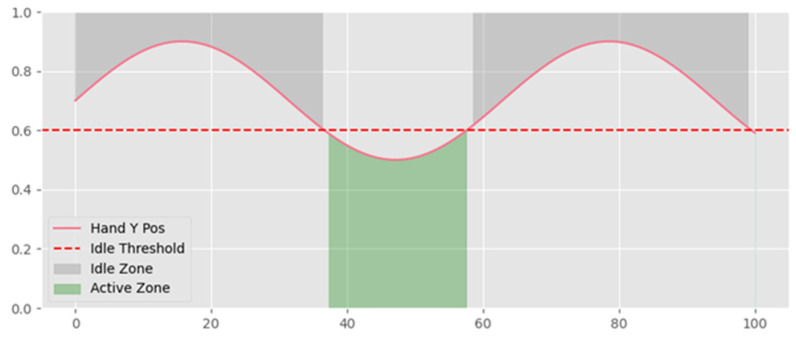
Hand-position dynamics illustrating the distinction between active signing and idle states.

To ensure that feature dimensions contribute comparably during training and to prevent dominant dimensions from biasing learning, landmark features are normalized using z-score normalization. The paper defines the normalization explicitly as shown in Equation (1):

(1) $$K_{norm}=\frac{K_{raw}-\mu }{\sigma }$$

where *K_raw_*, *µ*, and *σ* denote the raw landmark feature value, the mean, and standard deviation used for normalization from Equation (1), respectively. Because continuous signing streams do not provide explicit sentence boundaries, segmentation is a critical component of the pipeline. The proposed system uses idle-based segmentation by detecting transitions between signing activity and non-signing (idle) states. A conceptual view of the segmentation mechanism is provided in Figure 3, and an example illustrating the distinction between active signing and idle states is provided in Figure 4.

The paper specifies segmentation parameters selected to ensure stable detection under natural signing behavior, including:

- Wrist vertical position threshold: Y = 0.6 (normalized coordinate)
- Motion variance threshold: $\sigma^{2}$ = 0.02
- Idle duration window: 15 consecutive frames

An active signing state is detected when both hand wrist key-points exhibit Y-coordinates below the threshold (Y < 0.6) in conjunction with motion variance above the defined threshold, corresponding to raised hands during signing. Conversely, an idle state is identified when wrist positions remain at or above the threshold (Y ≥ 0.6) and motion variance remains below the idle threshold for at least 15 consecutive frames, corresponding to hands at rest. Both wrists are jointly evaluated during this process; if only one wrist satisfies the positional condition while the other does not, no state transition is triggered. This convention follows the standard MediaPipe image coordinate system, in which the origin is located at the top-left of the frame, and Y-values increase downward. Following segmentation, detected sentence clips can vary in duration. To standardize the input length for sequence modeling, temporal normalization is performed using linear interpolation–based resampling. The paper defines interpolation as shown in Equation (2):

(2) $$K_{\mathrm{interp}}(t)=K(t_{1})+\left[K(t_{2})-K(t_{1})\right]\frac{t-t_{1}}{t_{2}-t_{1}}$$

It also defines uniform temporal resampling to a fixed-length sequence (80 frames) as shown in Equation (3):

(3) $$t_{\mathrm{sampled}}=i\frac{T-1}{79},i\in \{0,1,\ldots ,79\}$$

where *T* denotes the original sequence duration in frames. The sampled indices span the full interval $\left[0,\;T-1\right]$. When indices are non-integer, values are obtained using linear interpolation according to Equation (2).

### 4.3. Sentence-Level Classification Network

**Figure 5:**
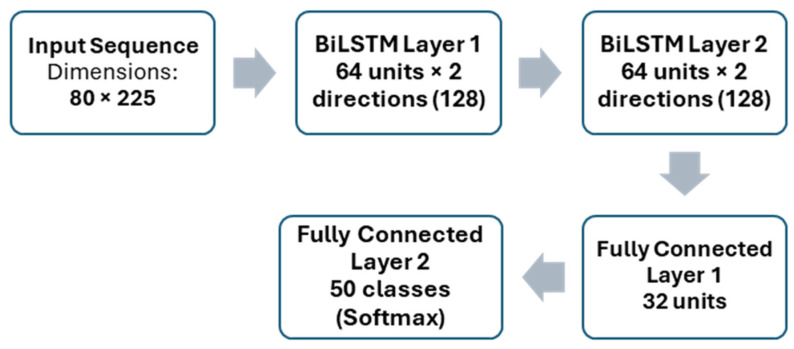
Architecture of the BiLSTM-based sentence classification network.

After temporal normalization, the fixed-length landmark sequence is passed to a BiLSTM network for sentence-level classification. Bidirectional modeling is used to exploit contextual dependencies across the full sentence sequence, which is particularly important for resolving ambiguities in continuous signing dynamics. The paper describes the network as consisting of two stacked BiLSTM layers, followed by fully connected layers and a softmax output layer. The architecture is shown in Figure 5. The paper defines the softmax probability computation as shown in Equation (4):

(4) $$P({\mathrm{class}}_{i})=\frac{e^{z_{i}}}{\sum_{j}e^{z_{j}}}$$

where $z_{i}$ denotes the logit corresponding to class i.

### 4.4. Post-Recognition Enhancement

The output of the BiLSTM classifier corresponds to a predicted sentence label based solely on visual input. Although this output is sufficient for quantitative evaluation, raw predictions may lack grammatical fluency when presented directly to end users. To address this limitation, an optional post-recognition enhancement stage is incorporated. This stage applies a lightweight language refinement model that reformulates the predicted sentence to improve readability while preserving semantic content. Importantly, this refinement is applied strictly as a post-processing step and does not influence recognition accuracy or any reported quantitative metrics in this paper. The refined sentence may be converted into spoken Arabic using a TTS module. Both refinement and speech synthesis are executed asynchronously to avoid introducing latency into the recognition pipeline.

## 5. Experimental Setup

This section presents the experimental design adopted to evaluate the proposed Arabic CSLR system. Beyond listing parameter settings, this section aims to justify the experimental choices, particularly with respect to robustness to signer variability, temporal inconsistency, and real-time operation.

### 5.1. Training Protocol and Experimental Design

The experimental evaluation is designed to rigorously assess the system’s ability to generalize to unseen signers, ensuring that the model learns signer-invariant temporal dynamics rather than identity-specific motion cues. Prior to dataset partitioning, all continuous signing streams are segmented via the idle-detection mechanism and normalized to a fixed temporal length of 80 frames. This pre-processing step effectively standardizes input dimensionality for the recurrent network while strictly preventing temporal leakage between training and testing subsets. By enforcing a signer-independent protocol where samples from the same individual never appear simultaneously in both sets, the evaluation provides a realistic measure of performance in practical, open-world deployment scenarios.

To facilitate controlled hyperparameter optimization and ablation analysis, a Fixed Signer-Independent Split was established as the primary validation benchmark. In this configuration, Signer 6 was held out exclusively for testing, while the data from the remaining five signers were partitioned into training and validation subsets. The training/validation partition followed a 70% to 30% split on the data of Signers 1 to 5, while Signer 6 served as the held-out test set. This fixed split provides a stable benchmark for controlled architectural comparison and analysis in Section 6.1, Section 6.2, Section 6.3 and Section 6.4.

To further examine robustness across the signer population and assess generalization to unseen individuals, a signer-disjoint Leave-One-Signer-Out (LOSO)-based evaluation framework was adopted. The dataset comprises six signers; therefore, six evaluation folds were constructed. In each fold, one signer was held out exclusively for testing, a second distinct signer was designated as the validation set for model selection and early stopping, and the remaining four signers were used for training. This rotation was repeated such that each signer served exactly once as the test signer and once as the validation signer across the six folds. Data augmentation was applied exclusively to the training subset, while validation and test subsets remained unaltered. Early stopping was guided solely by performance on the validation signer, ensuring that model selection was not influenced by identities seen during training. Final performance was computed on the unseen test signer for each fold, and overall accuracy is reported as the mean and standard deviation across folds.

Model training is performed using the Adam optimizer with a fixed learning rate of 0.001 and categorical cross-entropy loss. Training is conducted for up to 50 epochs; however, optimization typically terminates earlier due to early stopping based on validation loss. Early stopping monitors validation loss computed on the designated validation signer after each epoch, and training continues as long as improvement is observed within a patience window of five epochs. If validation loss fails to improve for five consecutive epochs, training is terminated, and the model parameters corresponding to the minimum validation loss are restored for final evaluation on the held-out test signer. Consequently, the number of epochs visible in the learning curves reflects the epoch at which early stopping occurred rather than the predefined maximum training limit. Early stopping acts as a regularization mechanism, preventing overfitting while ensuring that reported results correspond to a model state that generalizes well to unseen data. The complete set of training and optimization hyperparameters is summarized in Table 2. A batch size of 32 sequences is used consistently across all experiments. Dropout with a probability of 0.2 is applied within the BiLSTM layers to mitigate co-adaptation of recurrent units, which is particularly important given the relatively limited number of distinct sentence classes.

### 5.2. Evaluation Metrics and Measurement Rationale

System performance is evaluated primarily using sentence-level classification accuracy. This metric directly reflects the task objective of correctly recognizing complete signed sentences, rather than individual frames or isolated signs. Frame-level or gloss-level metrics are not reported, as they do not adequately capture the semantic correctness of sentence-level predictions in continuous signing scenarios. To complement overall accuracy and account for potential class imbalance, the weighted F1-score is also reported for LOSO comparisons. By weighting class-wise precision and recall according to sample frequency, this metric provides a more informative assessment of performance across all sentence categories. Per-class accuracy is analyzed to identify sentence classes that are consistently recognized and those that exhibit higher confusion rates. This analysis supports the qualitative error investigation presented later and helps attribute performance degradation to visual similarity or overlapping motion patterns between sentences. Real-time performance is assessed through inference latency and throughput measurements. Latency is defined as the model inference time required to classify a pre-processed, standardized sentence segment (80 frames) using the BiLSTM network. Throughput is reported as segment inference rate (inferences/s), computed over standardized 80-frame segments to quantify the system’s ability to sustain continuous real-time operation.

### 5.3. Implementation Details, Reproducibility, and Fairness

All experiments were conducted using a modular computational pipeline separating training and deployment environments. Landmark extraction and final real-time inference were performed on a local workstation equipped with an AMD Ryzen 5 5500U processor (6 physical cores, 12 threads) and 16 GB RAM. All reported latency and throughput measurements correspond to CPU-only execution without GPU acceleration. During inference, PyTorch 2.1.0 utilized all available CPU cores via its default OpenMP backend. Model training, validation, and cross-validation were conducted in the Kaggle cloud environment using an NVIDIA Tesla T4 GPU (16 GB GDDR6 memory) to ensure stable convergence across folds.

## 6. Results

This section presents a detailed experimental evaluation of the proposed real-time continuous SSL sentence-level recognition system. The experimental results are organized to examine complementary aspects of system performance. Section 6.1, Section 6.2 and Section 6.3 present quantitative performance analyses obtained under the fixed signer-independent evaluation setting. Section 6.4 evaluates real-time deployment characteristics, while Section 6.5 reports results under the signer-disjoint LOSO protocol. Section 6.6 presents additional diagnostic analyses derived from six-fold signer-disjoint training runs.

### 6.1. Robustness of Idle-Based Temporal Segmentation Parameters

A critical challenge in CSLR lies in the reliable detection of sentence boundaries within unsegmented signing streams. The proposed system addresses this challenge through an idle-based temporal segmentation mechanism. To ensure that reported performance is not dependent on fragile parameter tuning, a systematic sensitivity analysis was conducted. Table 3 reports segmentation performance metrics. The results reveal a broad operating region in which performance remains stable, with segmentation accuracy degrading gradually rather than abruptly outside the optimal configuration. This behavior indicates that the segmentation mechanism is robust to moderate parameter variation and does not rely on precise threshold calibration. Such robustness is essential for real-world deployment, where signer speed, camera placement, and environmental conditions may vary.

The segmentation mechanism is implemented as a deterministic idle-state detection process designed to maintain stable behavior under real-time operating conditions. The robustness results in Table 3 indicate reliable boundary detection across a broad parameter range.

### 6.2. Sentence-Level Recognition (SLR) Accuracy and Confusion Analysis (Fixed Signer-Independent Evaluation)

**Figure 6:**
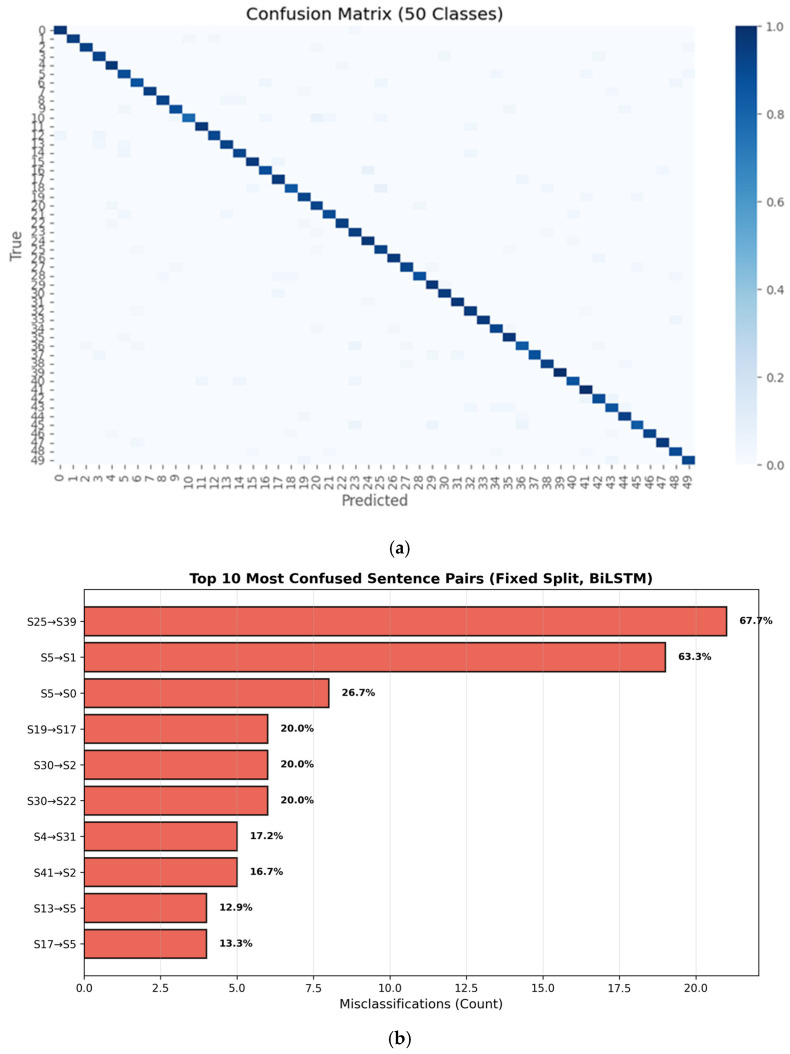
Sentence-level confusion analysis of the proposed BiLSTM model under signer-independent evaluation across all 50 sentence classes: (**a**) Normalized confusion matrix; (**b**) Top 10 most frequently confused sentence pairs (denoted as True Class or Predicted Class). The annotated percentages represent the proportion of true class instances that were misclassified as the predicted class.

The overall SLR performance is summarized in Table 4. The proposed system achieves high accuracy and balanced precision-recall performance under signer-independent evaluation, confirming the effectiveness of direct sentence-level temporal modeling. To analyze the structure of residual errors, the full sentence-level confusion matrix across all 50 classes is presented in Figure 6a, which demonstrates strong diagonal dominance and the scarcity of widespread or systematic class confusion. For clarity, Figure 6b summarizes the ten most frequently confused sentence pairs derived from the full confusion matrix. No dominant confusion clusters are observed, indicating that residual errors arise primarily from intrinsic visual similarity between certain sentence patterns rather than systematic segmentation failures or model instability. This localized error behavior supports the adequacy of the chosen landmark-based temporal representation for discriminating sentence-level dynamics under fixed signer-independent evaluation.

### 6.3. Effect of Sentence Length and Temporal Complexity

To assess whether the model captures genuine temporal dependencies rather than static pose cues, recognition performance under the fixed signer-independent evaluation was analyzed as a function of sentence length and motion complexity. Table 5 reports accuracy stratified across increasing levels of temporal complexity. The mean confidence denotes the average of the maximum softmax probability across all test samples in the corresponding sentence-length category. The results indicate consistently high accuracy for short and moderately complex sentences, with graceful degradation as sentence length increases. This trend demonstrates that the BiLSTM effectively models long-range temporal dependencies, although performance naturally decreases as sequences become longer and more variable. Notably, the absence of abrupt performance collapse suggests that the model does not overfit to short sequences and maintains meaningful temporal abstraction as complexity increases.

The observed accuracy reduction for longer sentences reflects increasing temporal variability and co-articulation effects commonly encountered in continuous signing sequences. This behavior highlights the growing importance of long-range temporal modeling as sequence duration increases. The implications of this behavior for different sequence modeling architectures are further examined in Section 7.

### 6.4. Real-Time Performance and Deployment Considerations

**Figure 7:**
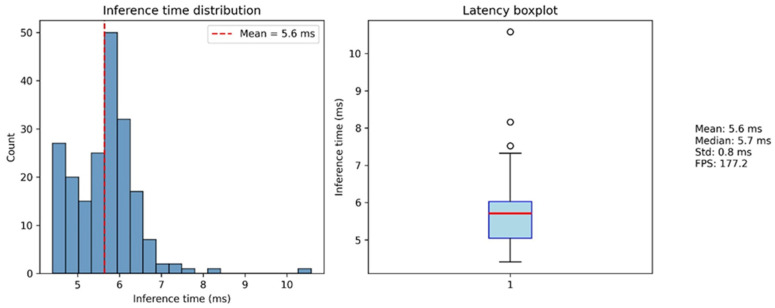
Distribution of sentence-level inference latency during real-time operation.

Beyond recognition accuracy, practical deployment requires stable real-time performance under limited hardware resources. Figure 7 illustrates the distribution of sentence-level inference latency during live execution. The latency profile exhibits a tightly bounded distribution with low variance around the mean, with only a few infrequent deviations. These results demonstrate stable and predictable runtime characteristics, supporting the suitability of the proposed system for real-time interactive applications.

**Figure 8:**
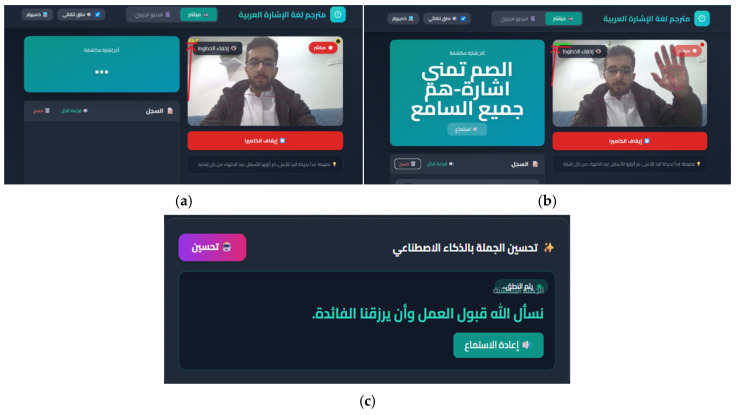
Qualitative demonstration of the real-time system: (**a**) input video with landmark extraction, (**b**) raw BiLSTM sentence-level predictions, and (**c**) LLM-refined output with TTS synthesis. ***Note:*** All identifiable individuals shown in system screenshots are authors of this work and have provided explicit informed consent for publication.

The qualitative system behavior during live operation is illustrated in Figure 8. The figure shows the actual steps of live video input with MediaPipe landmark overlays, idle-based segmentation indicating active and idle states, raw BiLSTM predictions with confidence scores, and the final LLM-refined Arabic sentence with TTS output. While aggregate metrics provide a concise summary of real-time performance, they do not capture runtime variability under continuous operation.

### 6.5. Signer-Independent LOSO Evaluation

To quantify the impact of recurrent architecture choice on signer-independent performance, Table 6 reports a direct comparison between BiLSTM, uni-directional LSTM, and GRU models under the same LOSO evaluation protocol. As shown in the table, the BiLSTM achieves the highest mean sentence-level accuracy (0.942) as well as the highest macro-averaged and weighted F1-scores. More importantly, the BiLSTM exhibits substantially lower standard deviation across all metrics compared to LSTM and GRU, indicating more stable generalization across different signers. In contrast, both LSTM and GRU show noticeably higher variance, suggesting increased sensitivity to signer-specific execution styles. These results demonstrate that bidirectional temporal modeling provides a measurable advantage in both accuracy and robustness for continuous sentence-level SLR.

**Figure 9:**
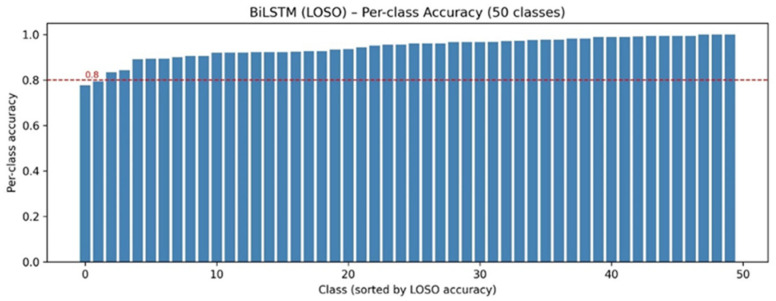
Per-class SLR accuracy of the proposed BiLSTM model under signer-independent LOSO evaluation. Sentence classes are sorted by accuracy to illustrate performance distribution and worst-case class behavior across the vocabulary.

To assess performance consistency across the sentence vocabulary, per-class recognition accuracy is shown in Figure 9, which indicates that most sentence classes are recognized with consistently high accuracy, and that no individual class exhibits severe performance degradation. Table 7 reports the test accuracy, macro-averaged F1-score, and weighted F1-score for each held-out signer, along with the mean and standard deviation across all signers. The proposed model achieves consistently high performance across all LOSO folds, with a mean accuracy of 0.942 ± 0.008, indicating limited sensitivity to signer identity. The close agreement between macro-averaged and weighted F1-scores across signers further suggests balanced performance across the sentence vocabulary rather than dominance by frequent classes. More importantly, no individual signer exhibits a pronounced degradation, despite differences in sample counts, supporting the conclusion that the landmark-based sentence-level modeling approach captures signer-invariant temporal dynamics. These LOSO results provide strong empirical evidence that the system generalizes reliably to previously unseen signers, reinforcing its suitability for practical deployment scenarios.

### 6.6. Training Behavior Analysis Under Six-Fold Signer-Disjoint Runs

**Figure 10:**
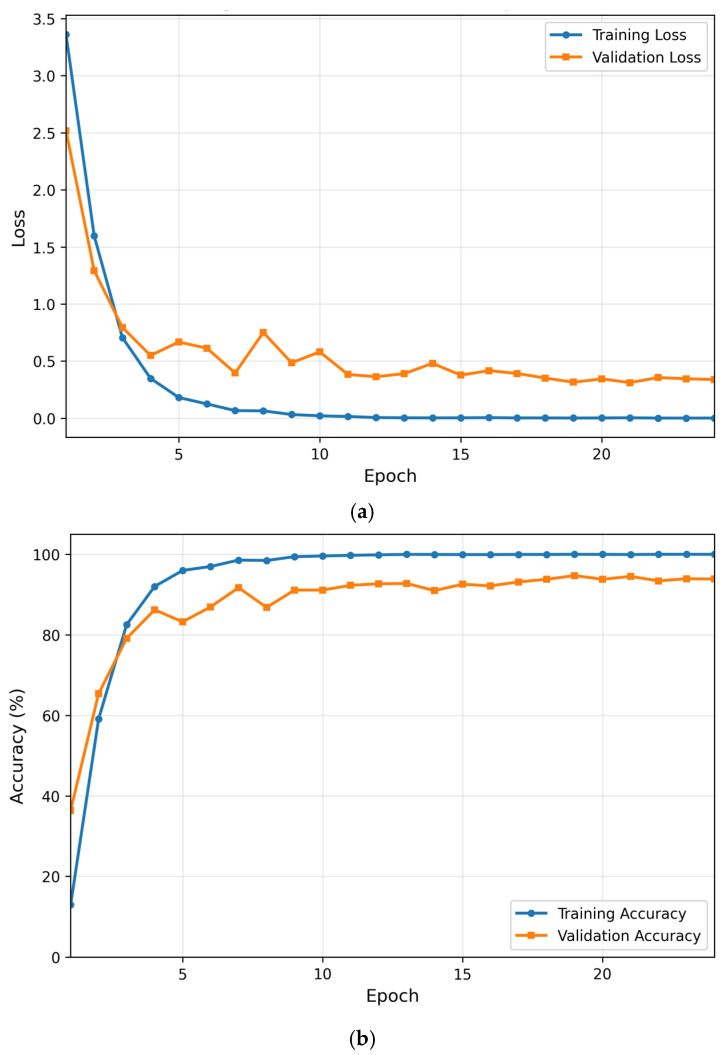
Training and validation dynamics obtained from a representative fold of the six-fold signer-disjoint training runs: (**a**) Loss and (**b**) Accuracy.

The optimization behavior of the BiLSTM classifier is analyzed using training dynamics obtained from a representative fold of the six-fold signer-disjoint training runs, as illustrated in Figure 10. Since validation performance is computed on a signer not included in the training set, the observed convergence behavior reflects generalization to unseen signer dynamics rather than memorization of identity-specific motion patterns. The loss curves exhibit smooth, monotonic decay without oscillatory behavior or divergence, indicating that the chosen learning rate and regularization strategy are well matched to the temporal classification task. Importantly, the close tracking between training and validation loss suggests that the model is not overfitting to signer-specific motion patterns. Similarly, the accuracy curves converge to nearly identical plateaus, demonstrating that performance gains during training translate consistently to unseen signers. This behavior suggests that the model learns signer-invariant temporal representations rather than memorizing individual signing styles. Error characteristics derived from the same six-fold training runs are examined next through confusion analysis.

**Figure 11:**
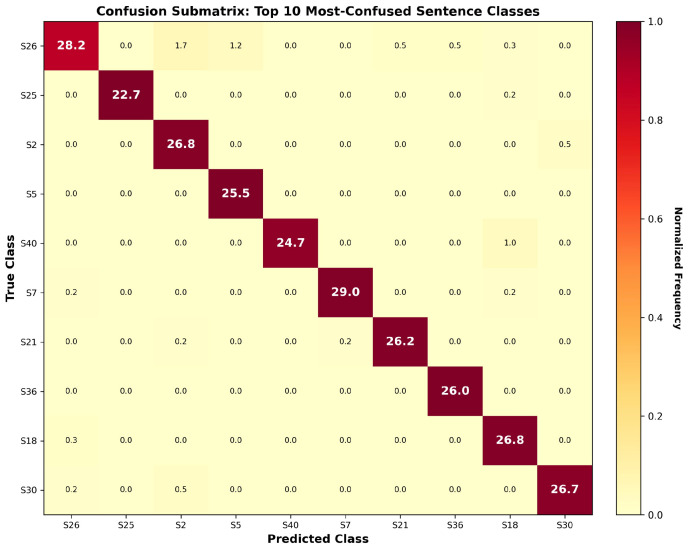
Confusion submatrix highlighting the ten most frequently confused sentence classes within the six-fold signer-disjoint training runs.

To further examine error characteristics within the six-fold signer-disjoint training runs, Figure 11 presents the confusion submatrix for the ten most challenging sentence classes. Similarly to the fixed-split results, misclassifications remain sparse and are primarily confined to visually or temporally similar sentence pairs, with cross-class confusion probabilities remaining low across all examined classes. The absence of dominant confusion clusters suggests that residual errors in the six-fold training runs arise primarily from similarities in motion dynamics rather than from systematic model bias.

**Figure 12:**
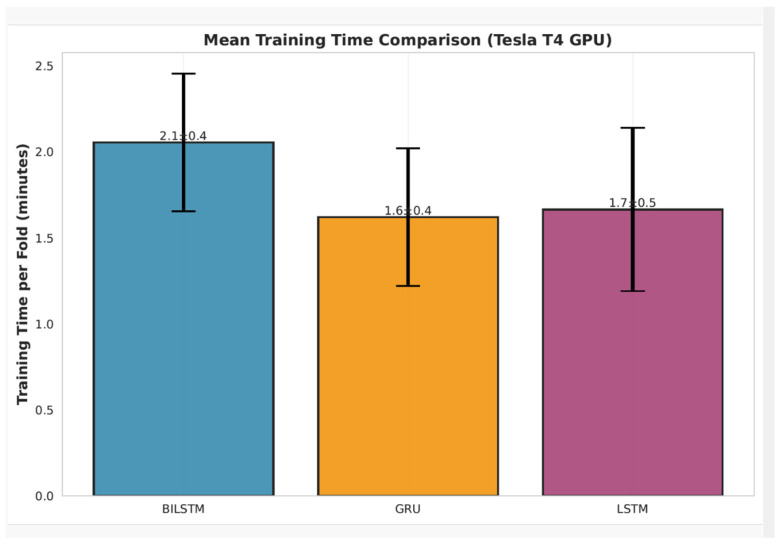
Mean training time per fold in the six-fold signer-disjoint training runs for the recurrent architectures (BiLSTM, LSTM, and GRU), measured on an NVIDIA Tesla T4 GPU.

To quantify computational training cost, the mean training time required to complete one fold of the six-fold training runs was measured for each evaluated recurrent architecture under identical hardware conditions, as summarized in Figure 12.

## 7. Discussion

The experimental results demonstrate that the proposed system achieves robust and reliable SLR of continuous SSL under real-time constraints. On the fixed signer-independent split (Signer 6 held out), the system achieved a baseline accuracy of 92.07%, establishing a strong performance benchmark. More importantly, under the LOSO evaluation protocol, the model demonstrated a superior mean sentence-level accuracy of 94.2%. This improvement under comprehensive cross-validation indicates that the model effectively captures signer-invariant temporal dynamics of continuous signing and generalizes well to entirely new individuals.

A principal achievement of this study is the validation of direct sentence-level modeling as a viable alternative to gloss-based pipelines for Arabic SLR. By operating on complete sentence segments rather than decomposed intermediate units, the system avoids cumulative error propagation associated with segmentation and gloss recognition stages. The comparative analysis against gloss-level baselines highlights a substantial performance gap, reinforcing the practical advantage of end-to-end sentence modeling for continuous ArSL, especially when signer-independent generalization is required.

The idle-based segmentation mechanism plays a critical enabling role in this performance. The stable recognition behavior observed across live deployment and offline evaluation suggests that sentence boundaries are reliably detected without explicit user intervention. Error analysis further indicates that most misclassifications arise from intrinsic visual similarity between certain sentence pairs rather than segmentation failures. This suggests that the segmentation strategy successfully isolates semantically complete sentence executions, allowing the classifier to focus on modeling gesture content rather than compensating for boundary errors. The ability to accommodate natural pauses and variable signing speed is essential for realistic deployment and contributes directly to the system’s robustness.

The use of landmark-based representations extracted via MediaPipe proves effective in mitigating inter-signer variability. The close alignment between training and validation curves, together with consistently high per-class accuracy across the full sentence set, indicates that the learned representations capture signer-invariant motion patterns. By abstracting away appearance-dependent cues such as texture, color, and background, the system reduces sensitivity to individual differences in hand shape, clothing, and lighting conditions. This abstraction is a key factor in achieving stable performance under signer-independent evaluation and supports the suitability of landmark-based features for real-time Arabic SLR. The residual errors are concentrated among a small number of visually similar sentence pairs that share overlapping hand trajectories or initial configurations. There is no systematic confusion or mode collapse observed, and misclassifications remain sparse relative to the full 50-class space. This localized error behavior suggests that further performance gains are more likely to arise from future integration of enhanced modeling of subtle temporal or non-manual cues, such as fine-grained facial expressions, rather than from fundamental changes to the recognition framework.

From a deployment perspective, the system satisfies the latency requirements for interactive use. With a mean inference latency of 5.6 ms per standardized 80-frame sentence segment under CPU-only execution, the model operates well within the time budget of real-time video pipelines, corresponding to an effective processing capacity of approximately 177 sentence segments per second. The tight latency distribution observed during live execution indicates stable runtime behavior, with variability primarily attributed to sentence duration and temporal normalization rather than model execution itself. The asynchronous execution of post-recognition language refinement and TTS synthesis ensures that improvements in output fluency do not compromise recognition latency, which preserves responsiveness in user-facing scenarios.

Beyond aggregate performance metrics, it is also important to consider architectural trade-offs between recognition accuracy and computational efficiency when selecting a model for practical deployment. To examine this, Table 8 presents a controlled diagnostic comparison of multiple sequence modeling architectures conducted using a single LOSO fold, in which different models were evaluated under identical data conditions. It is important to note that this table is intended to provide qualitative insight into relative architectural behavior and efficiency characteristics, rather than to establish population-level performance, which is already reported using full LOSO evaluation.

The BiLSTM architecture exhibits a favorable balance between recognition performance and computational efficiency when compared to both unidirectional recurrent models and higher-capacity attention-based architectures. Across recurrent variants, the BiLSTM consistently outperforms the standard LSTM and GRU models, underscoring the importance of bidirectional temporal context for sentence-level SLR. While the Transformer model attains higher raw accuracy in this single-fold diagnostic setting, it does so with substantially increased model size, requiring more than twice the number of parameters compared to the BiLSTM. The inference latency differences across architectures remain modest, indicating that the primary trade-off lies in model size rather than execution speed. In addition, in this diagnostic, the BiLSTM exhibits lower latency variability (±0.02 ms) than the Transformer (±0.10 ms), indicating more consistent runtime behavior under the same evaluation conditions. Taken together, these observations support the selection of the BiLSTM as a well-balanced compromise between accuracy and efficiency for real-time deployment on resource-constrained platforms. This trade-off prioritizes latency stability and data efficiency over peak accuracy, which is essential for real-time deployment on constrained vocabularies.

Despite the strong deployment performance and architectural efficiency demonstrated by the proposed system, the scope of the current study has some limitations. Future work may explore adaptive or learned segmentation strategies for larger-scale datasets or less controlled signing environments. The dataset is restricted to a controlled indoor environment and a fixed vocabulary of 50 ArSL sentences, and performance under more diverse environmental conditions or open-vocabulary settings remains unexamined. Additionally, while landmark-based representations capture core signing dynamics effectively, certain linguistic nuances conveyed through facial expressions are not captured, as the current system focuses exclusively on manual and somatic features. These limitations define a realistic boundary for interpreting the reported results and provide a useful foundation for future extensions.

## 8. Conclusions and Future Work

This paper presented a real-time, end-to-end system for sentence-level recognition and translation of continuous ArSL, with Saudi Sign Language (SSL) considered as a primary deployment target. The proposed approach integrates landmark-based feature extraction, idle-based temporal segmentation, and bidirectional recurrent modeling to directly recognize complete sign language sentences without reliance on gloss annotations or manual segmentation. Experimental evaluation on the ArabSign dataset under a LOSO protocol demonstrates that the system achieves a mean recognition accuracy of 94.2% while maintaining low latency and stable real-time performance. The results confirm that direct sentence-level modeling is both effective and practically advantageous for continuous Arabic SLR, particularly in deployment scenarios where intermediate annotations are unavailable. The combination of landmark-based representations and idle-based segmentation enables robust handling of signer variability and natural signing behavior, while the asynchronous integration of language refinement and speech synthesis enhances output usability without compromising responsiveness.

Future work will focus on extending the system beyond the current fixed-sentence vocabulary toward larger and more diverse linguistic coverage, including open-vocabulary and domain-specific signing scenarios. Expanding data collection to include a broader range of signers, environments, and lighting conditions will be essential for improving robustness and generalization. Further investigation into richer modeling of non-manual features, as well as the integration of additional modalities where appropriate, may help resolve remaining ambiguities among visually similar sentences. Finally, the development of a fully featured and user-friendly interface will support broader deployment and facilitate real-world adoption of the proposed system in educational, public service, and everyday communication contexts.

## Figures and Tables

**Table 1:** Summary of the Arabic continuous sign language dataset used for sentence-level recognition.

Item	Value
Total videos	9335
Sentence classes	50
Signers	6
Total duration	10 h 13 m
Average frames per sentence	≈87
Frame rate	24 FPS

**Table 2:** Training and optimization hyperparameters.

Parameter	Value
Optimizer	Adam
Learning rate	0.001
Batch size	32
Maximum epochs	50
Dropout rate	0.2
Sequence length	80 frames
Feature dimension	225
Early stopping patience	5 epochs

**Table 3:** Sensitivity and robustness analysis of idle-based segmentation parameters.

Wrist Y-Threshold (Normalized)	False Activation Rate	Missed Sentences	Avg. Segmentation Accuracy
0.4	18.7%	2.1%	79.2%
0.5	8.3%	5.4%	86.1%
0.6	3.2%	1.8%	92.4%
0.7	1.1%	8.7%	90.2%
0.8	0.2%	15.3%	84.5%

**Table 4:** Overall sentence-level recognition performance metrics. Note: While not a direct benchmark on identical data, the performance gap between the gloss-based Isharah baseline (≈60%) [26] and the proposed sentence-level approach (92%) highlights the inherent difficulty of gloss-based segmentation errors versus the robustness of direct sentence modeling.

Method	Recognition Level	Dataset	Evaluation Protocol	Accuracy
**Gloss-based recognition model**	Gloss/word level	Isharah	Signer-dependent	55–65% [26]
**Proposed BiLSTM [This work]**	Sentence level	ArabSign	Signer-independent	92.07%

**Table 5:** Recognition accuracy stratified by sentence length and temporal complexity (fixed split).

Sentence Length	Number of Signs	Test Accuracy	Mean Confidence
Short	1–2 signs	94.2%	0.847
Medium	3–4 signs	92.1%	0.803
Long	5+ signs	88.3%	0.762
Overall	1–5+ signs	92.07%	0.802

**Table 6:** Performance comparison of recurrent architectures under signer-independent LOSO evaluation on the ArabSign dataset.

Model	Accuracy (Mean ± Std)	Macro-F1 (Mean ± Std)	Weighted-F1 (Mean ± Std)
**BiLSTM**	0.942 ± 0.008	0.9400 ± 0.0097	0.9389 ± 0.0095
**LSTM**	0.9355 ± 0.0194	0.9289 ± 0.0242	0.9269 ± 0.0250
**GRU**	0.9354 ± 0.0230	0.9299 ± 0.0282	0.9285 ± 0.0287

**Table 7:** Signer-independent LOSO performance of the proposed BiLSTM model on the ArabSign dataset. Results are reported for each held-out signer, with the mean and standard deviation computed across all held-out signers.

Signer ID	No. of Samples	Accuracy	Macro-F1	Weighted-F1
**1**	1533	0.946	0.944	0.944
**2**	1600	0.958	0.958	0.956
**3**	1640	0.937	0.935	0.931
**4**	1550	0.932	0.930	0.930
**5**	1500	0.937	0.930	0.930
**6**	1512	0.941	0.939	0.940
	9335 (Total)			
**Mean ± Std**		0.942 ± 0.008	0.940 ± 0.0107	0.938 ± 0.0104

**Table 8:** Diagnostic comparison of sequence modeling architectures under a single LOSO fold (one held-out signer). Training GPU memory refers to peak memory usage during model training on a Tesla T4 GPU. Inference latency is measured under CPU-only execution.

Architecture	Test Accuracy	Inference Latency (ms)	Model Parameters	Training GPU Memory (GB)
BiLSTM	94.24%	5.51 ± 0.02 ms	254 K	0.06
Transformer	97.29%	5.63 ± 0.10 ms	635 K	0.03
LSTM	93.55%	4.96 ± 0.01 ms	112 K	0.05
GRU	93.54%	4.89 ± 0.02 ms	85 K	0.05

## Data Availability

The original contributions presented in this study are included in the article. Further inquiries can be directed to the corresponding author.
